# Expanded carrier screening for autosomal recessive conditions in health care: Arguments for a couple‐based approach and examination of couples' views

**DOI:** 10.1002/pd.5437

**Published:** 2019-02-28

**Authors:** Mirjam Plantinga, Erwin Birnie, Juliette Schuurmans, Anne H. Buitenhuis, Elise Boersma, Anneke M. Lucassen, Marian A. Verkerk, Irene M. van Langen, Adelita V. Ranchor

**Affiliations:** ^1^ Department of Genetics University Medical Center Groningen, University of Groningen Groningen The Netherlands; ^2^ Clinical Ethics and Law, Faculty of Medicine University of Southampton Southampton UK; ^3^ Department of Health Psychology University Medical Center Groningen, University of Groningen Groningen The Netherlands; ^4^ Department of Internal Medicine University Medical Center Groningen, University of Groningen Groningen The Netherlands

## Abstract

**Background:**

Expanded carrier screening (ECS) is aimed at detecting carrier states for autosomal recessive (AR) or X‐linked conditions in couples from the general population planning a pregnancy. ECS is currently usually offered on an individual basis despite the fact that, for AR conditions, only carrier couples are at risk of affected offspring. In this paper, we present a couple‐based ECS test‐offer for AR conditions, where results are offered as couple‐results only, and describe how couples view such an offer.

**Methods and results:**

An online survey covering attitudes, perceived difficulty, and intention to take up couple‐based ECS was used to examine couples' views. Results show that in 76% of the participating couples there is no objection at all towards receiving couple‐results only. Most couples display similar views. Observed discrepancies usually involved one of the couple members having a positive view, whilst the other was neutral. Although views stayed strikingly stable after discussion, the partner's opinion was regarded as important in deciding whether or not to have testing.

**Conclusion:**

This study shows that most couples do not object to receiving couple rather than individual ECS results, have similar views towards the offer, and are able to discuss differences in views and intentions.

## Abstract 2

What's already known about this topic?

- Expanded carrier screening (ECS) has become widely available
- ECS is usually offered on individual basis, and individual carrier states are reported
- Views of potential individual users have been researched

What does this study add?

- A couple‐based ECS test‐offer for autosomal recessive conditions, where results are offered as couple‐results only
- An examination of couples' views towards this offer: most couples do not object to receiving couple‐results only

## 1 INTRODUCTION

Expanded carrier screening (ECS) has become widely available.^1^, ^2^, ^3^, ^4^, ^5^ It is aimed at detecting carrier states for autosomal recessive (AR) or X‐linked conditions in couples from the general population planning a pregnancy, with the aim to enhance reproductive choice. Several studies have demonstrated the contribution of ECS to this aim.^6^, ^7^, ^8^

Currently, ECS is usually offered on individual basis, and individual carrier states are reported. This individual‐based approach stems historically from offering preconception or premarital carrier screening to high‐risk groups. Examples are the offer of Tay Sachs disease screening to Ashkenazi Jews^9^ or other populations with high prior risk of individuals being carriers.^10^ We believe that the sequential screening and reporting of individual carrier states that has been common practice in the carrier screening offer to high‐risk groups is no longer helpful or necessary when switching to population‐based ECS for AR conditions. In this paper, we present a couple‐based ECS test‐offer for AR conditions, where results are offered as couple‐results only, and describe how couples view such an offer.

Knowledge of individual carrier status for AR conditions only has reproductive utility if we know the status of the other partner. Given that the risk of being a carrier of a particular condition included in the ECS offer is generally low, individual carrier status does very little to predict offspring risk. Reporting of individual carrier states with the aim of cascade screening of family members is, in the context of ECS, therefore of little value. It is only the positive “couple‐result” which conveys increased risk for future offspring. Disclosing individual carrier status therefore has no clear advantage, whilst it may lead to anxiety and perceptions of illness^11^, ^12^ and goes together with high cost of follow‐up testing. Lynch et al examined the time needed to provide genetic counselling in the context of preconception carrier screening.^13^ They found that 78% of study participants were carriers of at least one condition and that the median time for results disclosure was 64 minutes (range 5‐229 min). Whilst some have expressed concern that individual carrier states are important in case couples split up^2^, ^14^, we argue that also in this situation individual carrier status has little reproductive value. In case couples split up, and the new couple wishes to have a child, a new couple‐based ECS test can be done with the new partner.

Also, the argument that people have the right to receive individual carrier states does not apply. Because no individual carrier states are being generated by our analysis, there are no results being withheld. The European General Data Protection Regulation (GDPR) act does grant data subjects the right to receive, free of charge, the personal data they have previously provided in a “commonly used and machine readable format” (https://eugdpr.org/). The GDPR act thus grants couples who have taken an ECS test the right to receive the raw data that is generated, but not the interpretation of this data in terms of individual carrier status.

The University Medical Center Groningen (UMCG) has developed a population‐based ECS test for implementation in a public health care system. The test is couple‐based, meaning that a couple receives a result based on their combined results; no individual carrier states are reported. The test screens for ∼70 genes associated with 50 very serious early‐onset AR conditions for which no treatment is currently available to alter the long‐term outcome. For couples in the Dutch general population, the chance of being a carrier couple for one of the conditions included in the test is approximately 1 in 150.^15^ The selection criteria and composition of the UMCG ECS panel have been described elsewhere.^16^ See also Supporting Information. Given that our ECS test is couple‐based, deciding whether or not to have this test is not an individual matter, but a joint decision made by the couple. Exploring how couples view the offer of such a test is therefore important. To our knowledge, there is little previous literature on couples' views and intentions regarding ECS testing. Two studies reported on couples' views on preconception carrier screening for single conditions, but in those studies members of the couple also received their individual carrier status. Becker et al^17^ reported on couples' views regarding preconception screening for Tay‐Sachs disease, and Henneman et al^18^ did the same for cystic fibrosis. Both studies found that both partners held similar views and concluded that decisions about participation in testing could be predicted more accurately by using a couple's combined view.

The current study aims to investigate couples' views on couple‐based ECS testing. We examined how couples view a couple‐based ECS test‐offer; to what extent partners hold similar views and have similar intentions, where the similarities/discrepancies lie, and what the size is of any discrepancy. We further described how much respondents' views and intentions changed after they had discussed the ECS test‐offer with their partner.

**What's already known about this topic?**

- Expanded carrier screening (ECS) has become widely available
- ECS is usually offered on individual basis, and individual carrier states are reported
- Views of potential individual users have been researched

**What does this study add?**

- A couple‐based ECS test‐offer for autosomal recessive conditions, where results are offered as couple‐results only
- An examination of couples' views towards this offer: most couples do not object to receiving couple‐results only

## 2 METHODS

### 2.1 Sample and survey design

**Figure 1:**
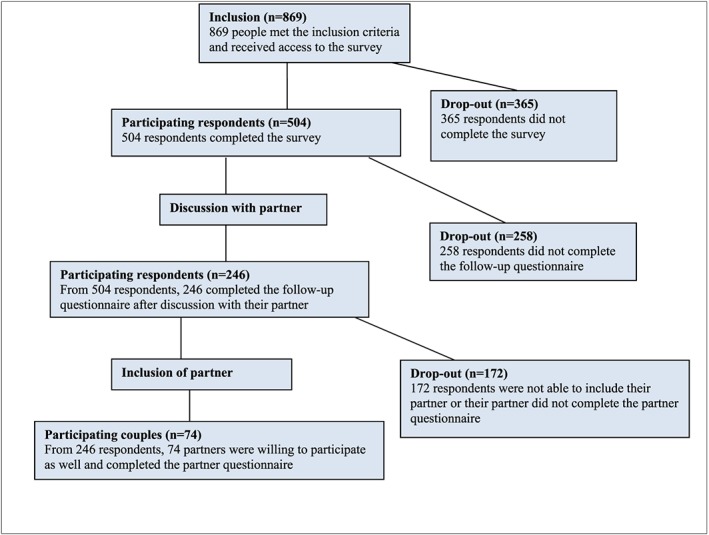
Flow chart of the study design [Colour figure can be viewed at wileyonlinelibrary.com]

This study is part of a larger study on potential users' views and intentions towards couple‐based ECS and framing of information, for which participants from the general population were recruited online. The study design has been published elsewhere.^16^, ^19^ Potential participants, women, and men of reproductive age (18‐40 years of age) with a different‐sex partner, were recruited online by a survey research sampling company (Survey Sampling International, SSI; http://www.surveysampling.com) in March 2014. SSI panel participants were invited to participate in our survey, and sampling was stratified according to sex, educational level, and geographical region in order for the sample to be representative for the Dutch population. Only participants who met all inclusion criteria were given access to the online questionnaire. A total of 869 individuals met the inclusion criteria and received access. Of these, 504 (58%) respondents completed the survey. The study's flow diagram is depicted in Figure 1.

Study participants were asked to fill in three questionnaires: T0 (before framing of information), T1 (after framing of information, before discussion), and T2 (after discussion). Potential participants were invited until we had 500 respondents who matched the abovementioned inclusion criteria and completed T0 and T1. The results of individual participants on T0 have been described in Plantinga et al^16^ and the effects of the framing of information between T0 and T1 in Voorwinden et al.^19^ After completing T0 and T1, respondents were asked to (1) invite their partners to also participate in the study, (2) discuss couple‐based ECS with their partners, and (3) after discussion, fill in questionnaire T2. The partners were asked to fill in one questionnaire independent from the other member of the couple after discussing couple‐based ECS. This study presents the results of each respondent and her/his partner. Ethical clearance for the study was granted by the Medical Ethical Review Committee of the UMCG (M14.152635).

### 2.2 Measures

#### 2.2.1 Sociodemographic characteristics

The following sociodemographic characteristics were recorded: sex, age, religion, and educational level. Educational level was categorized as: “low” (finished primary school, lower secondary school or vocational training); “intermediate” (higher level secondary school or intermediate vocational training); and “high” (higher vocational training or university).

#### 2.2.2 Relationship characteristics

We included duration of relationship (in years), relationship satisfaction, and the expressed desire to have children with this partner. Relationship satisfaction was measured on a 10‐point scale from 1 (very unsatisfied) to 10 (very satisfied).^20^, ^21^

#### 2.2.3 Views on the offer of a couple‐based ECS test

In exploring couples' views on the couple‐based ECS test‐offer, we included four measures based on the Theory of Planned Behaviour framework.^22^ *Attitude towards the couple‐based ECS test‐offer* was measured by the initial response towards the couple‐based ECS test‐offer. Answers were rated on a 7‐point Likert scale with anchors very negative (1) and very positive (7). *Objection towards receiving couple‐results only* was measured by asking participants whether they objected to the communication of couple‐results only (yes/no/do not know). *Perceived difficulty of decision* was measured with one item, referred to as perceived behavioural control in the Theory of Planned Behaviour framework^22^, and asked how difficult the person found the decision whether or not to have the couple‐based ECS test. This item was rated on a 5‐point Likert scale with anchors very difficult (1) and very easy (5). *Intention to have the couple‐based ECS test* was measured with one item from the Theory of Planned Behaviour framework^22^, “If this test were to be offered, I would be willing to participate”, and rated on a 7‐point Likert scale with anchors unlikely (1) and likely (7).

For the analyses, the individual scores were categorized as negative/neutral/positive. In doing this, 5‐point Likert scales were categorized as “negative” (scores 1‐2), “neutral” (3), and “positive” (4‐5) and 7‐point Likert scales were categorized as “negative” (scores 1‐2), “neutral” (3‐5), and “positive” (6‐7).

#### 2.2.4 Couples' discussions about the couple‐based ECS test‐offer

To gain understanding of couples' discussions on the couple‐based ECS test‐offer, we included six measures. *Duration of discussion* was measured by asking couples how long they spent discussing the couple‐based ECS test with their partner, measured in five 10‐minute intervals from <10 minutes to >40 minutes. *Topics discussed* were measured by listing 14 likely topics (eg, first reaction, risks, ethical aspects, conditions included in the test, consequences of the included conditions for parents and future child, reproductive options, decision to have ECS) and asking couple members to score each topic they had discussed in five time intervals (not discussed; <5 minutes; 5 minutes; 10 minutes; >10 minutes). *Satisfaction with discussion* was measured by a scale of three items (Cronbach's α 0.92), how pleasant, easy, and calm the discussion was rated, with a 5‐point Likert scale (anchors displeasing/pleasant, difficult/easy, turbulent/calm). The average single‐item scores were combined into a 1‐5 composite score and categorized as negative (1‐2), neutral (3), and positive (4‐5). *Perceived difficulty of discussion* was measured on a 5‐point Likert scale (anchors very difficult [1] and very easy [5]). The scores were categorized as difficult (1‐2), neutral (3), and easy (4‐5). *Perceived importance of partners' opinion* was measured on a 5‐point Likert scale with anchors not very important (1) and very important (5). The scores were categorized as not important (1‐2), neutral (3), important (4‐5). *Perceived influence of discussion* was measured by two items: to what extent participants felt they had influenced their partner's opinion and to what extent their opinion had been influenced by their partner. Answers were rated on a 5‐point Likert scale (anchors not at all [1] and very much [5]). The scores were categorized as little (1‐2), neutral (3), and much (4‐5).

### 2.3 Data analysis

Sociodemographic characteristics, relationship characteristics, and couples' views on ECS were described with N (%) for nominal and ordinal variables and mean (SD) or median (IQR) for interval and ratio variables. Differences between respondents with or without follow‐up measurement and with or without included partner (see Supporting Information) were tested using the chi‐square test for nominal/ordinal variables, with the unpaired Student's t‐test for interval/ratio variables with approximately normal distributions and with the nonparametric Mann‐Whitney U test for interval/ratio variables with skewed distributions.

As mentioned before, respondents from both sexes were recruited first and completed questionnaires both before and after having had a discussion about couple‐based ECS with their partner. The partners were recruited at a later point in time and only filled in one questionnaire, after discussion. To examine couples' combined views and similarities and discrepancies within couples, we compared respondent's T2 (measured *after* discussion) and partner's T0 (measured *after* discussion), except for the variable *attitude towards receiving couple‐results only*. Because this variable has only been measured in respondents' T0 (measured *before* discussion), we could only compare the variable at this time‐point with the partners' T0 measurement (measured *after* discussion). Changes in individual respondents' views after discussion with their partner were based on the comparison of T0 (before discussion) and T2 (after discussion). We chose to use T0 instead of T1, because T0 measures respondents' initial reaction and the study of Voorwinden et al^19^ showed that the manipulation between T0 and T1 did not affect respondents' scores.

Discrepancies in respondents' views before and after their discussion were quantified as N (%) and tested with the Stuart‐Maxwell Test for paired data. Changes were labelled as “large” if a change involved a change from a negative to a positive position or vice versa. Changes were labelled as “small” if a change involved a change from or towards a neutral position. Changes in views between male and female respondents were compared univariately with Fisher's Exact tests. In all analyses, a *P*‐value < .05 (two‐sided) was considered as statistically significant. Analyses were performed using IBM SPSS version 22 (IBM Corp., Armonk, NY, USA).

## 3 RESULTS

### 3.1 Respondent characteristics

Of the 504 recruited respondents, 246 respondents (49%) had a discussion with their partner and completed the follow‐up questionnaire (Figure 1). Of these 246 participating respondents, 172 of their partners did not complete the partner questionnaire, leaving 74 couples (30%) with complete data for comparison. Table 1 presents the respondent characteristics of our sample (see Supporting Information for a comparison of our sample with the drop‐out).

**Table 1:** Sociodemographic characteristics and views of respondents

	Respondents with before/after measurement (n = 246)	Respondents with before/after measurement and participating partner (n = 74)
Sociodemographic characteristics (score range)		
Respondent's sex (% female)	186 (76%)	52 (70%)
Age (in years; range 18‐40)	27 (24‐34)	29 (24‐34)
Religious (% yes)	85 (35%)	32 (43%)
Educational level		
Low	20 (8%)	9 (12%)
Intermediate	127 (52%)	36 (49%)
High	99 (40%)	29 (39%)
Relationship characteristics		
Duration relationship (in years; range 0‐25)	5.2 (2.8‐8.7)	6.3 (3.2‐9.6)
Relation satisfaction (1‐10)	8 (7‐10)	9 (8‐10)
Wish to have child		
Yes	175 (71%)	56 (76%)
No	56 (23%)	15 (20%)
Already pregnant	15 (6%)	3 (4%)
Views towards couple‐based ECS		
Attitude towards couple‐based ECS (1 = negative, 7 = positive)	5.2 (1.2)	5 (4‐6)
Objection towards receiving couple‐results only (% positive)	187 (76%)	58 (78%)
Difficulty of decision to take the test (1 = difficult, 5 = easy)	3.1 (1.0)	3.2 (1.0)
Intention to take the test (1 = likely; 7 = unlikely)	3 (2‐4)	3 (2‐5)

The respondents participating in this study can be described as being mostly female, having an intermediate to high education level, displaying high relation satisfaction and having a longer lasting relationship, indicating a stable relation, and having a positive attitude towards couple‐based ECS, although the decision to take up ECS is not perceived as being easy.

### 3.2 How couples view the couple‐based ECS test‐offer

#### 3.2.1 Similarity of views

**Figure 2:**
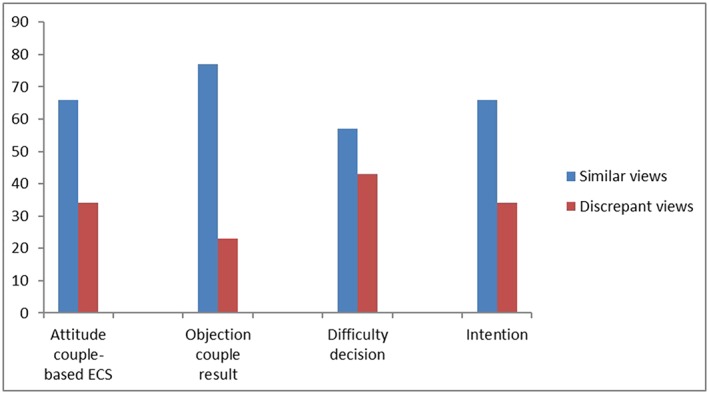
Similarity in views within couples (in % of total) (n = 74) [Colour figure can be viewed at wileyonlinelibrary.com]

Figure 2 shows, for each of the four included measures, the proportion of couples who had similar views (ie, both partners being positive, neutral, or both being negative). The similarity between couples was lowest for the perceived difficulty of the decision to have a couple‐based ECS test (57%) and highest for the objection towards receiving a couple‐result only (77%). In 66% of the couples, both couple members displayed a similar level of attitude towards couple‐based ECS and a similar level of intention.

#### 3.2.2 Evaluation of couple‐based ECS test‐offer by non‐discrepant couples

**Figure 3:**
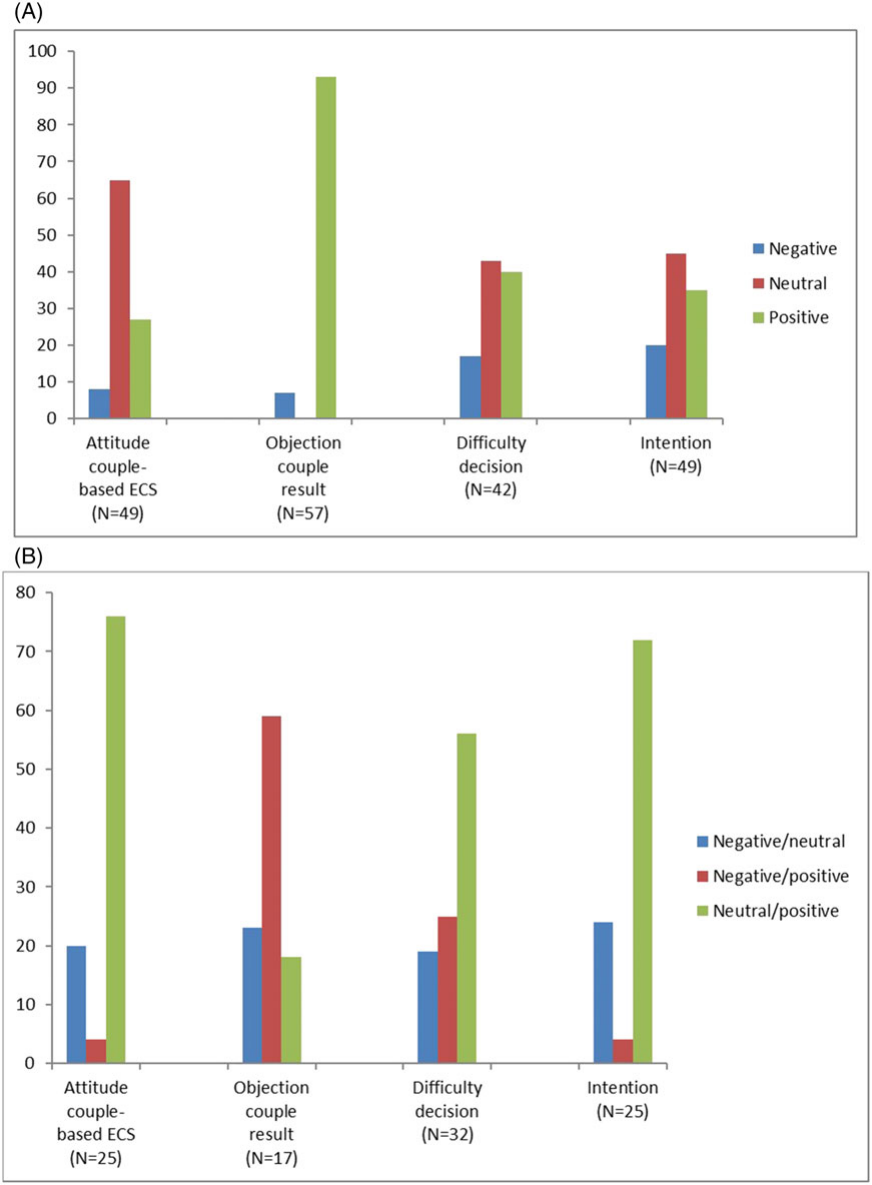
A, Evaluation of couple‐based ECS test‐offer (in % of total) by nondiscrepant couples; B, evaluation of couple‐based ECS test‐offer (in % of total) by discrepant couples [Colour figure can be viewed at wileyonlinelibrary.com]

Figure 3A shows how nondiscrepant couples (that is, couples in which both partners displayed the same view) evaluated the couple‐based ECS test‐offer. The data show that most couples do not object towards receiving a couple‐result only: 93% of the couples said they (both) did not object to this, whilst 7% (four couples) did. Most couples also do not perceive the decision to have couple‐based ECS as difficult: 40% perceive the decision as easy, 43% is neutral, and 17% perceive the decision as difficult. If the couple‐based ECS test were to be offered, both partners intended to have the test in 35% of the couples, in 20% both did not have the intention to participate, and in 45% of the couples both partners were neutral. Couples displayed most reservation in their attitude towards couple‐based ECS, measured by participants' initial response towards the couple‐based ECS test‐offer. In 65% of the couples, both partners displayed a neutral attitude, in 27% of the couples a positive attitude, and in 8% of the couples both partners had a negative initial response towards the couple‐based ECS test‐offer.

#### 3.2.3 Evaluation of couple‐based ECS test‐offer by discrepant couples

Figure 3B shows how discrepant couples (that is, couples in which both partners did not display the same view) evaluated the couple‐based ECS test‐offer.

Overall, we found that discrepancies within couples often involved one of the couple members displaying a positive view, whilst the other was neutral. An exception was seen on the objection towards receiving a couple‐result only, in which discrepancies in 59% (10 couples) of the cases involved one of the couple members displaying a positive view (not objecting), whilst the other was negative (objecting). Also, in 23% (four couples), one of the couple members was neutral, whilst the other was negative (objecting).

### 3.3 How respondents' views changed after discussion with their partner

#### 3.3.1 What topics did respondents discuss with their partner?

Respondents reported that discussion with their partner lasted less than 10 minutes in 31% of discussions, 60% lasted between 10 and 30 minutes, and 9% were >30 minutes. The topics discussed most often (in 87%‐88% of the couples) were (1) consequences for the affected child, (2) whether the couple wanted to have a couple‐based ECS test, and (3) consequences for the parents of having an affected child. In all cases, topics were discussed only briefly (<5 minutes by half of the couples). The respondents were overall positive about the discussion with their partner (75% positive, 23% neutral, 2% negative). Half of respondents (49%) perceived the couple‐based ECS test‐offer as an easy to discuss topic, 39% was neutral, and 12% found it difficult. In their decision to take up testing, most respondents (87%) considered their partners' opinion important, 9% were neutral, whilst 4% found it not important. The vast majority of the respondents felt that their discussions had little influence on their own decision to have testing (92% little, 7% neutral, 1% much) nor that it influenced their partner's decision much (88% little, 10% neutral, 2% much).

#### 3.3.2 How much did respondents' views change after discussion?

Finally, we examined whether respondents' views towards the couple‐based ECS test‐offer changed after discussion with their partner. Table 2 shows the differences, as well as their size (small/large) and direction (increase/decrease), of respondents' views both before and after discussion.

**Table 2:** Differences in attitudes and intentions before and after discussion in female and male respondents (in % of total)

	All (N = 246) (% of total)	Female (N = 186) (% of total)	Male (N = 60) (% of total)	Female vs Male (P‐Value)^a^
Attitude towards couple‐based ECS				0.75
Increase large	0	0	0	
Increase small	10	10	10	
No change	67	68	63	
Decrease small	23	22	27	
Decrease large	0	0	0	
Increase vs decrease (P‐value)^b^	0.00**	0.00**	0.03*	
Difficulty of decision				0.30
Increase large	2	2	2	
Increase small	19	19	18	
No change	55	56	53	
Decrease small	20	18	27	
Decrease large	4	5	0	
Increase vs decrease (P‐value)^b^	0.42	0.53	0.59	
Intention to take up couple‐based ECS				0.13
Increase large	1	1	0	
Increase small	11	10	13	
No change	75	74	82	
Decrease small	13	15	5	
Decrease large	0	0	0	
Increase vs decrease (P‐value)^b^	0.13	0.60	0.13	

a Fishers's Exact Test is used to compare differences in change in attitudes and intentions between female and male respondents.

b Stuart‐Maxwell Test is used to compare the increase with the decrease in attitudes and intentions in all respondents, female respondents and male respondents, respectively.

* *P* < 0.05.

** *P* < 0.01.

The results show that most respondents (55%‐75%) held similar views before and after discussion with their partner. Where changes were found, these could be in either direction, but overall the magnitude of the change was small (changing from neutral to positive or negative or vice versa). However, the attitude towards the couple‐based ECS test‐offer, measured as participants' initial response towards this test, was an exception: here, significantly more respondents (23%) reported a decrease in attitude than an increase in attitude (10%). This decrease mostly involved a change from a positive position before discussion towards a neutral attitude after discussion. The perceived difficulty of the decision to have the test changed most often: 45% of the respondents reported a change in perceived difficulty of the decision. The changes went, however, in both directions, equally for the men and women in the couples, implying that for some the decision was easier after discussion, whilst for others it was more difficult. Male and female respondents did not differ significantly in this respect.

The intention to take up couple‐based ECS was most stable: 25% of the respondents reported a change in intention after discussion with their partner. The changes went, in both directions, equally for the men and women in the couples, implying that for some intention increased, whilst for others it decreased. Male and female respondents did not differ significantly in this respect. Finally, we examined whether changes in respondents' views were correlated with characteristics of the discussion that respondents had had with their partner. Table 3 displays the bivariate (Spearman) correlation coefficients between the different discussion characteristics and changes in attitude, perceived difficulty, and intention after discussion. None of the discussion factors is significantly correlated with a change in view after discussion.

**Table 3:** Bivariate (Spearman) correlation coefficients between discussion characteristics and changes in attitude, perceived difficulty, and intention after discussion (*N* = 246)

	Change in attitude towards couple‐based ECS^a^	Change in perceived difficulty of decision^a^	Change in intention to take up ECS^a^
Discussion duration (short = 1 ... Long = 3)	0.110 (0.84)	0.011 (0.866)	0.028 (0.667)
Discussion satisfaction (negative = 1 ... Positive = 3)	−.025 (0.692)	0.055 (0.395)	−.061 (0.338)
Perceived difficulty of discussion (difficult = 1 ... Easy = 3)	0.028 (0.667)	0.074 (0.250)	−.008 (0.907)
Importance of partners' opinion (not‐important = 1 ... Important = 3)	−.088 (0.171)	0.066 (0.304)	0.006 (0.925)
Perceived influence of discussion on own opinion (little = 1 ... Much = 3)	0.074 (0.247)	0.025 (0.700)	0.036 (0.577)
Perceived influence of discussion on opinion partner (little = 1 ... Much = 3)	0.043 (0.498)	0.012 (0.856)	0.013 (0.840)

a A larger score represents a larger change, regardless of the direction. Scale range is 0 to 5 for changes in attitude and change in intention, and 0 to 3 for change in perceived difficulty.

* *P* < 0.05.

** *P* < 0.01.

## 4 DISCUSSION

To our knowledge, this is the first report of couples' views towards the offer of an ECS test for AR conditions which reports couple‐results only. We examined how couple members view the offer of such a couple‐based ECS test in terms of the intention to have couple‐based ECS, the perceived difficulty of the decision, the initial response towards the test‐offer, and the objection towards receiving couple‐results only. We found that the offer of a test that does not report individual carrier status was not seen as problematic by the majority of couples: in 76% of all participating couples, there was no objection at all towards receiving couple‐results only, both partners of the couple did object in 5% of the couples, in another 5% of the couples one of the partners objected and the other was neutral, and, finally, in 14% of the couples one objected and one did not.

Henneman and Ten Kate^23^ investigated couples' disclosure preferences regarding CF screening and found that 94% of the participating couples preferred full disclosure, meaning receiving individual carrier status as well, mainly because they felt that no information should be withheld from them. The differences in findings between our study and that of Henneman and Ten Kate^23^ might be due to framing of the concept of results. When giving the choice to know “all” results or only some, one might be more likely to answer the former. Frame the couple‐result as the only result that will have implications for future children, then the answers might be different, as is shown in our study.

Given that, in the context of ECS for AR conditions, reporting of individual carrier states is of little value nor for the tested couple or for cascade screening of family members, we argue that a couple‐based approach is the most responsible approach to implement ECS as population screening. Moreover, an ECS couple‐based approach that includes screening for very serious AR conditions only also prevents the potential blurring between carrier screening and predictive genetic testing that is present in the offer of ECS panels including both AR as well as X‐linked and even autosomal dominant conditions.^5^ This does not imply that carrier screening in women for prevalent X‐linked conditions should not be offered. This could be offered on an individual basis and combined with the couple‐based ECS test‐offer for AR conditions. Recently, the Superior Health Council of Belgium issued recommendations on the responsible implementation of ECS in the health care system in which they also argue in case of AR conditions for communication of couple‐results only. It is stated that individual carrier status may be provided in addition, but not as a default and only if explicitly requested by (one or both of) the couple.^24^ In another consideration, Kirk et al argue that especially when screening a large number of variants, a couple‐based approach is preferable because it reduces the associated analysis and follow‐up counselling burden.^13^

Looking at the overall evaluation of a couple‐based ECS test‐offer, we found that the neutral and positive views prevailed among couple members. Furthermore, we found that couples displayed large similarities in views: often, both couple members being neutral and rarely both members being negative. Respondents' views also stayed strikingly stable after having discussed the ECS test‐offer with their partner. The observed changes were only few and mostly small. The initial response towards couple‐based ECS did, however, significantly change after discussion. This usually involved a change from a positive view to a neutral standpoint. It might be that these respondents did not take certain complexities into account in their initial response. In order to be able to address the issues playing a role in couples' views towards couple‐based ECS, more (qualitative) research is necessary.

We are aware that there may be a gap between intended and actual behaviour^25^, ^26^ and that our study examined a hypothetical rather than an actual offer. A further limitation is our high drop‐out rate: we had complete data from only 74 couples (30%) of the 504 recruited respondents. Another limitation is an overrepresentation of initial responses from female participants. More women (70%) than men (30%) were willing to participate and able to include their partner. Most couples are therefore characterized by a female respondent and a male partner. Our sample is further characterized by an overrepresentation of highly educated participants (40%). The sample is also characterized by couples with a long‐lasting relationship who reported their relationship to be good, presumably the couples who are more likely to be actively considering having children.

To summarize, our study sheds light on how participating eligible couples view a couple‐based ECS test for serious AR diseases and their intentions to have such a test together if it were to be offered. Given that the offer of couple‐based ECS testing is aimed at enhancing reproductive choice for couples^1^, ^27^ and that the test result impacts both partners, one would need couples to reach a joint decision whether or not to participate in testing. In view of the positive results reported here, the next obvious step is to study joint decisions when couple‐based ECS is actually offered in a health care setting.

## FUNDING STATEMENT

We thank the Department of Genetics for financial support (Charles Buijs grant).

## CONFLICT OF INTEREST

The authors declare no conflict of interest.

## Supporting information

Data S1: Supporting information

Data S2: Supporting information

Data S3: Supporting information
