# Supplementary material for: Expanded carrier screening for autosomal recessive conditions in health care: Arguments for a couple‐based approach and examination of couples' views
Source: Prenat Diagn. 2019 Feb 28;39(5):369–78. doi: 10.1002/pd.5437 (PMC6593986; doi:10.1002/pd.5437)
Supplement: Supplementary file 1 — Data S1: Supporting information [file PD-39-369-s001.docx]

**Supplementary Information**

**Criteria for conditions to be included in the UMCG ECS gene panel**

- Early-onset

- Characterized as very serious (severe mental and/or physical retardation, severe pain and/or premature death)

- Characterized as conditions for which no treatment is currently available to alter the long term outcome

**List of autosomal recessive conditions included in the UMCG gene panel**

| **Condition** | **Links** | **Gene** |
| --- | --- | --- |
| **Andermann Syndrome** | http://www.omim.org/entry/218000  http://www.orpha.net/consor4.01/www/cgi-bin/OC_Exp.php?lng=EN&Expert=1496 | SLC12A6 |
| **Aspartylglucosaminuria** | http://www.omim.org/entry/208400  http://www.orpha.net/consor/cgi-bin/OC_Exp.php?Expert=93 | AGA |
| **Ataxia Telangiectasia** | http://www.omim.org/entry/208900  http://www.orpha.net/consor/cgi-bin/OC_Exp.php?Lng=GB&Expert=100 | ATM |
| **Bloom Syndrome** | http://www.omim.org/entry/210900  http://www.orpha.net/consor/cgi-bin/OC_Exp.php?Lng=GB&Expert=125 | BLM (RECQL3) |
| **Canavan’s Disease** | http://omim.org/entry/271900  http://www.orpha.net/consor/cgi-bin/OC_Exp.php?lng=EN&Expert=141 | ASPA |
| **Citrullinemia type 1** | http://www.omim.org/entry/215700  http://www.orpha.net/consor4.01/www/cgi-bin/OC_Exp.php?lng=EN&Expert=247525 | ASS1 |
| **Congenital disorder of glycosylation type 1A** | http://www.omim.org/entry/212065  http://www.orpha.net/consor/cgi-bin/OC_Exp.php?Expert=79318 | PMM2 |
| **Congenital Nephrotic Syndrome, Finnish Type** | http://www.omim.org/entry/256300  http://www.orpha.net/consor/cgi-bin/OC_Exp.php?Expert=839 | NPHS1 |
| **D-Bifunctional Protein Deficiëncy** | http://www.omim.org/entry/261515 | HSD17B4 |
| **Epidermolysis Bullosa** | http://www.omim.org/entry/226700  http://www.omim.org/entry/226600 | LAMA3, LAMB3, LAMC2, COL7A1 |
| **Familial Dysautonomia** | http://www.omim.org/entry/223900  http://www.orpha.net/consor/cgi-bin/OC_Exp.php?Expert=1764 | IKBKAP |
| **GM2 Gangliosidosis, mainly Tay Sachs Disease** | http://www.omim.org/entry/272800  http://www.orpha.net/consor/cgi-bin/OC_Exp.php?lng=EN&Expert=845 | HEXA |
| **GRACILE Syndrome** | http://www.omim.org/entry/603358  http://www.orpha.net/consor/cgi-bin/OC_Exp.php?Expert=53693 | BCS1L |
| **Hypophosphatasemia** | http://omim.org/entry/241500  http://www.orpha.net/consor/cgi-bin/OC_Exp.php?lng=EN&Expert=436 | ALPL |
| **Infantile Sialic Acid Storage Disease** | http://www.omim.org/entry/269920  http://www.orpha.net/consor/cgi-bin/OC_Exp.php?Expert=834 | SLC17A5 |
| **Krabbe’s Disease** | http://www.omim.org/entry/245200  http://www.orpha.net/consor/cgi-bin/OC_Exp.php?lng=EN&Expert=487 | GALC |
| **Lipoamide Dehydrogenase Deficiëncy** | http://www.omim.org/entry/246900  http://www.orpha.net/consor/cgi-bin/OC_Exp.php?Lng=GB&Expert=2394 | DLD |
| **Leukoencephalopathy**  **with Vanishing White Matter** | http://www.omim.org/entry/603896  http://www.orpha.net//consor/cgi-bin/OC_Exp.php?Lng=GB&Expert=135 | EIF2B1, EIF2B2, EIF2B3, EIF2B4, EIF2B5 |
| **Metachromatic Leukodystrophy** | http://omim.org/entry/250100  http://www.orpha.net/consor/cgi-bin/OC_Exp.php?Expert=512 | ARSA |
| **Mitochondrial Recessive Ataxia Syndrome** | http://www.omim.org/entry/607459 | POLG |
| **Mitochondrial DNA depletion syndromes type 4A** | http://www.omim.org/entry/203700 | POLG |
| **Mitochondrial DNA Depletion Syndrome type 4 B** | http://www.omim.org/entry/613662 | POLG |
| **Mucolipidosis IV** | http://omim.org/entry/252650  http://www.orpha.net/consor/cgi-bin/OC_Exp.php?lng=EN&Expert=578 | MCOLN1 |
| **Mucopolysaccharidosis I (Hurler, Hurler-Scheie)** | http://omim.org/entry/607014  http://omim.org/entry/607015  http://www.orpha.net/consor/cgi-bin/OC_Exp.php?lng=EN&Expert=579 | IDUA |
| **Mucopolysaccharidosis III, Sanfilippo Syndrome** | http://omim.org/entry/252900  http://omim.org/entry/252920  http://omim.org/entry/252930  http://omim.org/entry/252940  http://www.orpha.net/consor/cgi-bin/OC_Exp.php?lng=EN&Expert=581 | SGSH, NAGLU, HGSNAT, GNS |
| **Mucopolysaccharidosis IV, Morquio Syndrome** | http://omim.org/entry/253000  http://omim.org/entry/253010  http://www.orpha.net/consor/cgi-bin/OC_Exp.php?lng=EN&Expert=582 | GALNS, GLB1 |
| **Mucopolysaccharidosis VI, Maroteaux-Lamy Syndrome** | http://omim.org/entry/253200  http://www.orpha.net/consor/cgi-bin/OC_Exp.php?lng=EN&Expert=583  http://www.maroteaux-lamy.com/en-gb/pages/hcp/index.aspx | ARSB |
| **Mucopolysaccharidosis VII, Sly Syndrome** | http://omim.org/entry/253220  http://www.orpha.net/consor/cgi-bin/OC_Exp.php?lng=EN&Expert=584 | GUSB |
| **Muscle Eye Brain Disease/ Muscular Dystrophy-Dystroglycanopathy (congenital with Brain and Eye Anomalies)** | http://www.omim.org/entry/253280  http://omim.org/entry/614643  http://omim.org/entry/615041 | POMGNT1; FKRP, ISPD, TMEM5, |
| **Neuronal Ceroid Lipofuscinosis, Types 1,2** | http://omim.org/entry/256730  http://www.orpha.net/consor/cgi-bin/OC_Exp.php?Expert=79263  http://omim.org/entry/204500  http://www.orpha.net/consor/cgi-bin/OC_Exp.php?Expert=168491 | PPT1, TPP1 |
| **Neuronal Ceroid Lipofuscinosis Type 3, Batten-Spielmeyer-Vogt’s Disease** | http://omim.org/entry/204200  http://www.orpha.net/consor/cgi-bin/OC_Exp.php?Expert=79264 | CLN3 |
| **Neuronal Ceroid Lipofuscinosis Type 5, Finnish Variant** | http://www.omim.org/entry/256731  http://www.orpha.net/consor/cgi-bin/OC_Exp.php?Expert=168491 | CLN5 |
| **Neuronal Ceroid Lipofuscinosis Type 8 & Progressive Epilepsy and Mental Retardation** | http://omim.org/entry/610003  http://www.orpha.net/consor/cgi-bin/OC_Exp.php?Expert=1947 | CLN8 |
| **Niemann-Pick’s Disease** | http://omim.org/entry/257220  http://www.orpha.net/consor/cgi-bin/OC_Exp.php?Expert=646  http://omim.org/entry/257200  http://www.orpha.net/consor/cgi-bin/OC_Exp.php?Expert=77292 | NPC1, SMPD1 |
| **Nijmegen Breakage Syndrome** | http://omim.org/entry/251260  http://www.orpha.net/consor/cgi-bin/OC_Exp.php?lng=EN&Expert=647 | NBN |
| **Osteopetrosis** | http://www.ojrd.com/content/4/1/5  http://omim.org/entry/259700  http://www.orpha.net/consor4.01/www/cgi-bin/OC_Exp.php?lng=EN&Expert=667 | TCIRG1 |
| **Osteogenesis Imperfecta, Type VII** | http://omim.org/entry/610682  http://www.orpha.net/consor/cgi-bin/OC_Exp.php?lng=EN&Expert=666 | CRTAP |
| **Polycystic Kidney Disease** | http://omim.org/entry/263200  http://www.orpha.net/consor/cgi-bin/OC_Exp.php?lng=EN&Expert=731 | PKHD1 |
| **Pontocerebellar Hypoplasia Type 1** | http://omim.org/entry/607596  http://www.orpha.net/consor/cgi-bin/OC_Exp.php?Expert=2254 | VRK1 |
| **Pontocerebellar Hypoplasia Type 2 (A,B & C)** | http://www.omim.org/entry/277470  http://www.omim.org/entry/612389  http://www.omim.org/entry/612390  http://www.orpha.net/consor/cgi-bin/OC_Exp.php?lng=EN&Expert=2524 | TSEN54, TSEN2, TSEN34 |
| **Pontocerebellar Hypoplasia Type 6** | http://omim.org/entry/611523  http://www.orpha.net/consor/cgi-bin/OC_Exp.php?lng=EN&Expert=166073 | RARS2 |
| **Rhizomelic Chondrodysplasia Punctata** | http://omim.org/entry/215100  http://omim.org/entry/222765  http://omim.org/entry/600121  http://www.orpha.net/consor/cgi-bin/OC_Exp.php?lng=EN&Expert=177 | PEX7, GNPAT, AGPS |
| **Sjögren-Larsson Syndrome** | http://omim.org/entry/270200  http://www.orpha.net/consor/cgi-bin/OC_Exp.php?lng=EN&Expert=816 | ALDH3A2 |
| **Spinal Musculaire Atrophy (SMA), type 0/1)** | http://omim.org/entry/253300  http://www.orpha.net/consor/cgi-bin/OC_Exp.php?lng=EN&Expert=70 | SMN1 |
| **Smith-Lemli-Opitz Syndrome** | http://omim.org/entry/270400  http://www.orpha.net/consor/cgi-bin/OC_Exp.php?Lng=GB&Expert=818 | DHCR7 |
| **Zellweger Syndrome** | http://omim.org/entry/214100  http://www.orpha.net/consor/cgi-bin/OC_Exp.php?Expert=912 | PEX1 |
