# Supplementary material for: Expanded carrier screening for autosomal recessive conditions in health care: Arguments for a couple‐based approach and examination of couples' views
Source: Prenat Diagn. 2019 Feb 28;39(5):369–78. doi: 10.1002/pd.5437 (PMC6593986; doi:10.1002/pd.5437)
Supplement: Supplementary file 2 — Data S2: Supporting information [file PD-39-369-s002.docx]

**Supplementary Information**

**Socio-demographic characteristics and views of respondents**

|  | *Respondents with before/after measurement*  *(n = 246)* | *Respondents without before/after measurement (n = 258)* | p-value | *Respondents with before/after measurement and participating partner (n = 74)* | *Respondents with before/after measurement without participating partner (n = 172)* | p-value |
| --- | --- | --- | --- | --- | --- | --- |
| **Socio-demographic characteristics**  **(score range)** |  |  |  |  |  |  |
| Respondent ’s sex (% female) | 186 (76%) | 178 (69%) | .10 (a) | 52 (70%) | 134 (78%) | .20 (a) |
| Age (in years; range 18-40) | 27 (24-34) | 28.5 (24-35) | .35 (c) | 29 (24-34) | 27 (23-34) | .66 (c) |
| Religious (% yes) | 85 (35%) | 90 (35%) | .94 (a) | 32 (43%) | 53 (31%) | .06 (a) |
| Educational level  low   intermediate   high | 20 (8%)  127 (52%)  99 (40%) | 43 (17%)  142 (55%)  73 (28%) | *.00* (a) | 9 (12%)  36 (49%)  29 (39%) | 11 (6%)  91 (53%)  70 (41%) | .31 (a) |
| **Relationship characteristics** |  |  |  |  |  |  |
| Duration relationship  (in years; range 0-25) | 5.2 (2.8 – 8.7) | 4.3 (2.2 – 7.1) | *.04* (c) | 6.3 (3.2-9.6) | 5 (2.7 – 8.3) | .06 (c) |
| Relation satisfaction (1 - 10) | 8 (7-10) | 8.5 (7-10) | .66 (c) | 9 (8-10) | 8 (7-9) | *.03* (c) |
| Wish to have child  Yes  No  Already pregnant | 175 (71%)  56 (23%)  15 (6%) | 176 (68%)  70 (27%)  12 (5%) | .45 (a) | 56 (76%)  15 (20%)  3 (4%) | 119 (69%)  41 (24%)  12 (7%) | .52 (a) |
| **Views towards couple-based ECS** |  |  |  |  |  |  |
| Attitude towards couple-based ECS (1= negative, 7= positive) | 5.2 (1.2) | 4.6 (1.3) | *.00* (b) | 5 (4-6) | 5 (4-6) | .59 (c) |
| Objection towards receiving couple-results only (% positive) | 187 (76%) | 167 (65%) | *.01* (a) | 58 (78%) | 129 (75%) | .57 (a) |
| Difficulty of decision to take the test (1= difficult, 5= easy) | 3.1 (1.0) | 3.0 (0.9) | .06 (b) | 3.2 (1.0) | 3.1 (1.0) | .27 (b) |
| Intention to take the test  (1= likely; 7= unlikely) | 3 (2-4) | 4 (2-5) | .11 (c) | 3 (2-5) | 3 (2-4) | .35 (a) |

ECS: Expanded Carrier Screening; Data in table represent: mean (SD) or median (Q1-Q3) or N (%) and were tested with (a) chi-square test; (b) unpaired Student’s t-test; (c) Mann-Whitney U test.
