# Supplementary material for: Expanded carrier screening for autosomal recessive conditions in health care: Arguments for a couple‐based approach and examination of couples' views
Source: Prenat Diagn. 2019 Feb 28;39(5):369–78. doi: 10.1002/pd.5437 (PMC6593986; doi:10.1002/pd.5437)
Supplement: Supplementary file 3 — Data S3: Supporting information [file PD-39-369-s003.docx]

**Supplementary Information**

**Survey items**

**Introduction Text**

We are all carriers of one or more hereditary diseases. Carriers do not have the disease but if you and your partner together are carrier of the same hereditary disease there is an enhanced chance that you will have a child with the disease you together are carrier of. The University Medical Centre Groningen is developing an expanded preconception carrier screening (ECS) test for couples for 50 serious hereditary diseases a child could inherit when both future parents are carriers.

We would like to ask you and your partner several questions regarding this subject to be able to receive a better understanding of your views about this testing opportunity. This makes it easier for us to adapt the test to the wishes of the users. All of your answers will be processed anonymously and are only accessible for the research team.

This EPCS test for couples is a new possibility for future parents to find out prior to a pregnancy if they are carrier of the same hereditary disease. This test is a blood test and gives clarity for 50 diseases. If you and your partner are both carrier of the same hereditary disease there is a 1 in 4 (25%) chance of having a child with the disease in *each* pregnancy. When you know you are a carrier couple for one of the serious inherited diseases included in the test, there are several options you can choose from to prevent having a child with the disease.

The questionnaire consists of two parts. You and your partner must answer the questions separately and not talk about it together. Some of the questions we will repeat. Please also answer these questions this is of high importance for the research. After answering the questions in part 1, you will have 5 days to discuss the information about the ECS test with your partner, before answering the questions of part 2. You will receive a reminder by email.

Thank you in advance for your cooperation.

To be clear: the **serious hereditary diseases** included in the test are all diseases that are untreatable, start early in life, are associated with severe pain, and/or severe physical disability, and/or severe mental disability, and/or early death.

**Attitude towards the couple-based ECS test-offer**

1. ***My initial response to his ECS test is***

| Very negative |  | | | | | Very positive |
| --- | --- | --- | --- | --- | --- | --- |
| - 1 | - 2 | - 3 | - 4 | - 5 | - 6 | - 7 |

**Objection towards receiving couple-results only**

1. ***The ECS test is currently only available for couples. Each couple receives a result based on their combined genetic information. Individual carrier results are not communicated. Do you object to the provision of the test to couples only?***

| - Yes |
| --- |
| - No |
| - I do not know/ I am not sure |

**Perceived difficulty of decision**

1. ***I find the decision whether or not to have the couple-based ECS test***

| - Very difficult |
| --- |
| - Difficult |
| - Not easy/not difficult - Easy - Very easy |

**Intention to have the couple-based ECS test**

1. ***If this ECS test were to be offered, I would be willing to participate***

| Unlikely |  | | | | | Likely |
| --- | --- | --- | --- | --- | --- | --- |
| - 1 | - 2 | - 3 | - 4 | - 5 | - 6 | - 7 |
